# Olfactory dysfunction in obesity and type 2 diabetes: mechanistic insights from preclinical models

**DOI:** 10.1007/s00125-026-06755-w

**Published:** 2026-05-30

**Authors:** Janice Bulk, Laura Casanueva Reimon, Sophie M. Steculorum

**Affiliations:** 1 Max Planck Institute for Metabolism Research, Max Planck Research Group Neurocircuit Wiring and Function, Cologne, Germany; 2 Excellence Cluster on Cellular Stress Responses in Aging-Associated Diseases (CECAD), University of Cologne, Cologne, Germany; 3 German Center for Diabetes Research (DZD), Neuherberg, Germany

**Keywords:** Diabetes, Energy homeostasis, Feeding, Glucose homeostasis, Hormones, Hypothalamus, Obesity, Olfaction, Review

## Abstract

Olfaction and its dysfunction have gained increasing interest across a broad spectrum of research areas including ageing and neurodegenerative and psychiatric disorders. In addition, olfactory dysfunction is increasingly recognised as a common feature of metabolic disorders, with mounting evidence linking an impaired sense of smell to obesity and type 2 diabetes. While the olfactory system was once considered negligible in humans, it has now emerged as a critical modulator of feeding-related behaviours, endocrine regulation, and energy and glucose homeostasis. Studies in both humans and animal models highlight the bidirectional interaction between olfactory processing and metabolic status, suggesting that olfactory changes are not merely consequences of obesity and type 2 diabetes, but may also contribute to their development and progression. This review summarises current findings on the mechanisms associated with olfactory dysfunction in obesity and type 2 diabetes in rodent models, from the initial detection of odorants in the nasal cavity to the downstream neural circuits and their physiological and behavioural outcomes. Particular emphasis is placed on the emerging concept of sensory regulation of metabolism, highlighting how food sensory cues, including food odours, influence whole-body metabolism and obesogenic responses. Finally, the therapeutic potential of targeting the olfactory system to improve olfactory performance and the olfactory regulation of whole-body metabolism is discussed. Olfactory-based therapeutics may offer novel and promising strategies for the prevention and treatment of metabolic diseases.

**Graphical Abstract:**

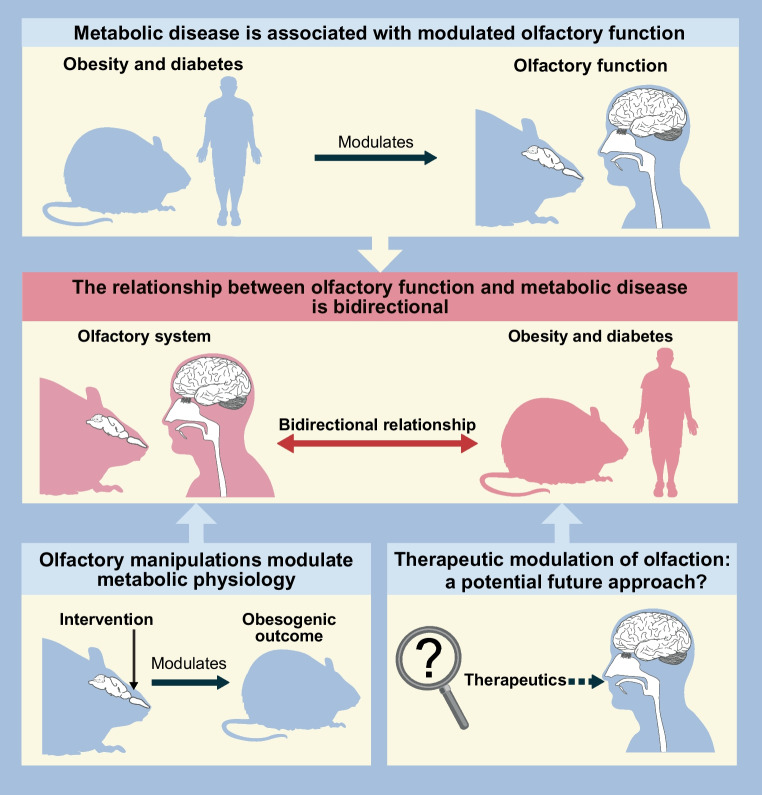

**Supplementary Information:**

The online version contains a slideset of the figures for download available at 10.1007/s00125-026-06755-w.

## Introduction

**Fig. 1:**
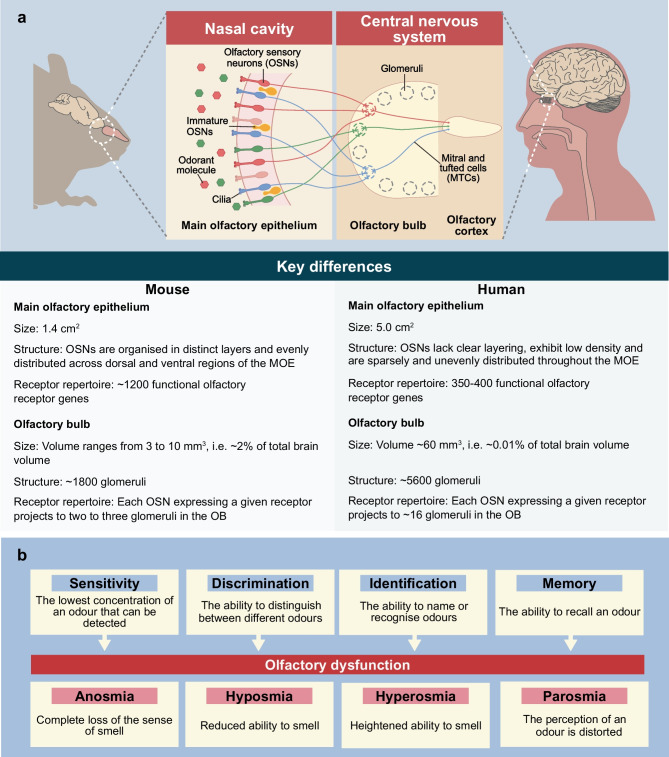
Visualisation of the similarities and differences between the olfactory systems in mice and humans. (**a**) The basic organisation of the olfactory system is conserved across mammals, including mice and humans; however, the olfactory system is proportionally more prominent in mice. In both species, odour molecules reach olfactory sensory neurons (OSNs), which are located in the main olfactory epithelium (MOE) of the nasal cavity. These OSNs are bipolar neurons that extend cilia into the nasal cavity to detect odours; they then send their axons to the olfactory bulb (OB), where they converge to form glomeruli. These glomeruli are innervated by mitral and tufted cells (MTCs), which relay olfactory information to higher brain regions, including the olfactory cortex. Odour activation can occur not only via the orthonasal route, but also retronasally in both humans and mice, albeit with less intensity and a delay compared with orthonasal olfaction. Structural and genetic differences distinguish the olfactory systems of mice and humans. These differences suggest that, despite having a smaller receptor repertoire, humans have more connections between each receptor type and glomerulus, potentially allowing greater combinatorial processing per receptor. As noted by Kikuta et al and McGann et al, human olfactory ability is not as poor as is commonly believed [1, 180]. Central image of the nasal cavity and central nervous system adapted from Sakano et al [181] with permission from Elsevier. (**b**) Olfactory function arises from multiple processes, including odour sensitivity (detection threshold), discrimination (the ability to differentiate between odours) and identification (recognition of odours) and olfactory memory. Impaired performance in one or more of these areas can contribute to clinical olfactory dysfunction, including quantitative alterations (anosmia, hyposmia and hyperosmia) and qualitative alterations (parosmia). For a detailed elaboration of olfactory function see Poirier and Melin [15]. This figure is available as part of a downloadable slideset

Olfaction has long been considered insignificant in humans, partly because the human olfactory system is relatively smaller than in other mammals (for a comparative view of rodent and human anatomy, see Fig. 1a) [1]. However, emerging scientific research has revealed the importance of the olfactory system in human physiology, reproduction and behaviour [1, 2] and its key role in shaping eating behaviour [3]. Emerging evidence has also demonstrated alterations in olfaction in a range of diseases including neurodegenerative, psychiatric and metabolic diseases such as obesity and type 2 diabetes [4, 5]. Obesity is a chronic, multifactorial disease characterised by excessive adipose tissue accumulation [6]. Obesity significantly increases the risk of several metabolic and cardiovascular conditions, including type 2 diabetes, a complex, chronic disease resulting from inadequate insulin secretion due to pancreatic beta cell dysfunction and/or a reduced cellular response to insulin [7]. Obesity and type 2 diabetes are accompanied by alterations in endocrine regulation and whole-body metabolism that are tightly linked to olfaction [8–11]. Although the causal relationship between impaired olfaction and metabolic dysregulation remains incompletely understood, compelling findings from rodent models suggest that olfactory processing might play a role in the development and progression of metabolic disorders, rather than being a mere consequence of metabolic alterations. In this review, we synthesise current evidence on olfactory alterations associated with obesity and type 2 diabetes, examining mechanisms from the earliest stages of nasal processing to resulting physiological and behavioural outcomes in rodent models. We further highlight olfaction’s role in modulating eating behaviour and discuss the therapeutic potential of targeting the olfactory system in metabolic disease.

## Olfactory changes in obesity and type 2 diabetes in humans

### Current evidence of olfactory impairments in obesity and type 2 diabetes

Increasing evidence supports a strong association between body weight and olfactory function, with individuals with a higher BMI showing an increased prevalence of olfactory dysfunction (Fig. 1b) [12–15]. In men, a high BMI correlates with altered olfactory function [13, 14] while, in women, visceral fat has been identified as the primary factor associated with olfactory dysfunction [16]. Interestingly, although increased BMI is generally associated with olfactory dysfunction, several studies have also reported enhanced sensitivity and lower detection thresholds specifically for food-related odours. Notably, satiated individuals with a high BMI exhibit higher olfactory discrimination for food-related odours [17] and individuals with obesity exhibit increased sensitivity to and preference for sweet caloric odours such as chocolate [18, 19]. The association of increased sensitivity and preference for food odours observed in obesity, despite an overall reduction in olfactory function, may contribute to enhanced food-driven feeding behaviour and a stronger preference for palatable diets. Further, bariatric surgery is linked to enhanced olfactory function [8, 13, 20, 21] regardless of diabetes status [20], suggesting that metabolic interventions can restore olfactory alterations.

Although not generally regarded as a diabetes-related complication [22], olfactory alterations have been documented in type 2 diabetes [12, 23, 24]. For instance, individuals with diabetes with complications demonstrate a higher prevalence of hyposmia and anosmia [25–27]. However, contrasting results have also been reported, demonstrating no significant differences in olfactory ability in individuals with diabetes [28]; this may be due to the low sample size in this study. Recent research indicates that metabolic impairments, that is, visceral fat and insulin resistance, rather than BMI alone are associated with reduced sensitivity to food odours, with insulin resistance negatively associated with olfactory function regardless of BMI [16, 18], underscoring the need for future studies to account for these variables.

Beyond changes in olfactory performance, a human neuroimaging study revealed differences in olfactory bulb (OB) volume between individuals with and those without obesity [29]. Functionally, the activation of the amygdala, which is a key hub linking olfactory, emotional and reward circuits, is increased in both genetic and non-genetic obesity [30, 31] and predicts food intake [32], suggesting its critical involvement in sensory regulation of feeding.

### Genetic variations affecting olfactory processing and metabolic disorders

Genetic variations in olfactory receptors and olfactory transduction pathway genes have been linked to obesity, eating behaviour and odour sensitivity [13, 33]. A genome-wide association study (GWAS) in Northern Han Chinese participants showed that the weighted genetic scores of the olfactory receptor family 4 subfamily D member 1 gene (*OR4D1*) and olfactory receptor family 52 subfamily K member 1 gene (*OR52K1*) are positively associated with obesity [34, 35], while the weighted genetic score of the olfactory receptor family 2 subfamily L member 8 gene (*OR2L8*) is negatively associated with obesity [34, 35]. In addition, a GWAS from the UK Biobank reported that dietary habits are associated with specific genetic loci [36]. Among the strongest associated loci, several regions contained olfactory receptor genes. Although loci associated with overall dietary patterns were not enriched for olfactory receptor genes, when specific foods were examined, several showed a clear association [36]. For example, the olfactory receptor family 6 subfamily B member 1 gene (*OR6B1*; SNP: rs10249294) shows a very strong association with a higher number of pieces of fresh fruit consumed per day [36]. Interestingly, several variants of the human olfactory receptor family 7 subfamily D member 4 gene (*OR7D4*) have been associated with changes in both dietary behaviour [37] and olfactory perception [38, 39]. One variant (SNP: rs2878329) has been linked to lower adiposity, reduced hunger and decreased cognitive dietary restraint [37]. In addition, two non-synonymous variants (rs61729907, R88W; rs5020278, T133M) that alter receptor function are associated with higher visceral adipose tissue area [37] and reduced sensitivity to androstenone, a testosterone-derived odorant present in male pig meat [38, 39]. Individuals carrying either one copy (RT/WM) or two copies (WM/WM) of these variants perceive androsterone as less intense and less unpleasant than individuals homozygous for the functional *OR7D4* (RT/RT) allele [38, 39], supporting the idea that genetic variation in olfactory receptors may connect olfactory perception with eating behaviour and obesity. Moreover, a specific polymorphism in the potassium voltage-gated channel subfamily A member 3 gene (*KCNA3*, also known as *KV1.3*; rs2821557), which is an insulin-sensitive voltage-gated potassium channel variant, has been associated with enhanced olfaction, resistance to diet-induced obesity (DIO) and protection against age-related olfactory decline, particularly in individuals homozygous for the major allele T variant [40, 41]. Altogether, while further research is needed to establish whether obesity-associated olfactory receptor variants are consistently linked to olfactory dysfunction, the evidence suggests that genetic variations in olfactory receptor genes might not only influence olfactory abilities but also modulate dietary choices, potentially influencing the development of obesity.

### The role of the nasal microbiota

The nasal cavity hosts a distinct microbiota essential for mucosal health and immune function [42], which is directly influenced by lifestyle factors, sex and nutritional status [42–44]. Variations in nasal microbes correlate with olfactory performance, with higher diversity linked to reduced function [43]. Notably, women with obesity exhibit increased nasal microbiota diversity [43, 45], suggesting a potential role in altered olfactory performance and metabolic diseases. However, further studies are necessary to clarify how microbial communities may contribute to olfactory dysfunction in obesity and type 2 diabetes.

## Decoding olfactory changes in obesity and type 2 diabetes: evidence from rodent models

While human studies provide valuable correlational observations, rodent models, given the conservation of the olfactory system across mammals, are key for studying metabolic and olfactory changes in obesity and type 2 diabetes to further delineate causal relationships.

### Olfactory alterations in diet-induced obesity models

Several studies have reported olfactory dysfunction in DIO models, with prolonged high-fat diet (HFD) exposure impairing odour discrimination, detection and memory formation [46–50], even after HFD withdrawal [48]. DIO decreases odour sensitivity and memory; however, its inconsistent effects on odour discrimination suggest a more complex regulation of olfactory function [46–51], likely influenced by odour selection, methodology and diet composition. DIO mice show impaired discrimination between unfamiliar distinct odorants [47, 48, 50, 51], such as flower extract [47, 50] or chemical odorants [48, 51], but not diet-related odours [49] or unfamiliar social scents [46], suggesting that olfactory impairments in obesity may not extend to familiar odours frequently encountered in the environment.

In addition, diet composition might also influence olfactory changes [46, 51, 52]. For instance, a Western-style diet (high in fat and sugar) [46] and fructose-enriched [51] diets impair odour learning [46], detection [46, 51] and discrimination [51]. However, highly processed diets impair olfactory function regardless of macronutrient composition [52], suggesting that other factors beyond diet composition or energy content shape obesity-related olfactory deficits in DIO mouse models, warranting further model refinement.

### Olfactory alterations in genetic models of type 2 diabetes and obesity

Beyond dietary intervention, genetic models can be used to investigate the contributions of obesity and type 2 diabetes to olfactory dysfunction. For example, in obesity-prone Sprague Dawley rats, genetic predisposition combined with a Western-style diet reduced insulin-mediated food-seeking, odour detection and olfactory learning [53]. Furthermore, rodents with deficient leptin production (*ob*/*ob* mice) or leptin receptor function (*db*/*db* mice, Zucker *fa*/*fa* rats) show increased olfactory sensitivity despite being obese [54, 55]. Moreover, leptin administration reduces food odour detection in *ob*/*ob* mice [55], confirming leptin’s regulatory role in olfaction and suggesting that obesity per se is not the primary driver of olfactory dysfunction in these obese genetic models. The Goto–Kakizaki rat, a polygenic lean spontaneous model of type 2 diabetes, has further disentangled the effects of insulin resistance from those of excess adiposity. Despite their lack of obesity, these rats have odour detection and memory deficits but intact odour discrimination [56], pointing to insulin resistance as a potential driver in olfactory impairment. In support of this, while some studies link olfactory changes to increased obesity and elevated serum leptin and insulin levels [49], others challenge this notion, showing that olfactory deficits can arise in the absence of obesity [47].

Genetic mouse models have helped to further elucidate the connection between olfaction and obesity. For example, *MC4R* knockout mice, a genetic mouse model of obesity in which mice lack a receptor involved in satiety, have been shown to exhibit impaired odour discrimination and failed to discriminate between two different odours [49], further underscoring the association between obesity and olfactory dysfunction. In contrast, whole-body knockout of *Kv1.3* resulted in enhanced odour discrimination even under HFD feeding [48, 49, 57, 58]. These ‘super-smeller’ mice were resistant to DIO, further highlighting that enhanced olfactory function may exert protective metabolic effects [48, 49, 57, 58]. Conversely, some studies have found an inverse link between olfactory function and obesity. For instance, disruption of IGF-1 signalling in olfactory sensory neurons (OSNs) in the main olfactory epithelium (MOE) led to enhanced olfactory function, yet increased adiposity and insulin resistance [59]. Further, ablation of OSNs in the MOE, resulting in hyposmia, protected mice from DIO and increased energy expenditure [59]. Collectively, these rodent studies underscore a complex, bidirectional relationship between metabolism and olfaction, raising the question of whether olfactory dysfunction is a cause or a consequence of obesity. Notably, enhanced olfactory sensitivity may exert opposing metabolic outcomes that may originate from the severity of the olfactory impairment (i.e. degree of hyposmia induced) and the anatomical specificity of the targeted deletion (i.e. MOE specificity). Future research should consider these variables to clarify olfaction’s role in energy balance and obesity.

These findings suggest that olfactory impairment can both result from metabolic changes and influence metabolic outcomes, highlighting the bidirectional nature of this relationship in rodent models. Importantly, while genetic rodent studies provide causal insights, human evidence is largely correlational, underscoring the need for translational studies to clarify causality in humans.

## Mechanistic insights into olfactory dysfunction in obesity and diabetes in rodents

### Disruption of the MOE in obesity and type 2 diabetes

**Fig. 2:**
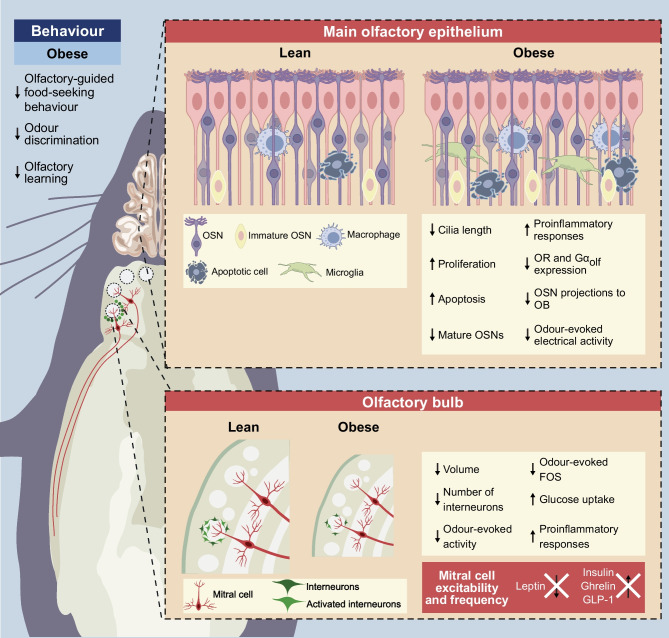
Influence of obesity and type 2 diabetes on the olfactory system. Rodent studies have shown that obesity alters olfactory function, reducing olfactory-guided food-seeking behaviour and impairing odorant discrimination and olfactory learning. A high-energy diet in rodents disrupts homeostasis in the MOE, resulting in increased apoptosis and cellular proliferation, a reduction in the number of mature OSNs and elevated infiltration of microglia and macrophages. OSNs have been reported to have shorter cilia and reduced expression of olfactory receptors (ORs) and Gα_olf_ in obese rodents. A reduction in OSN projections to the olfactory bulb (OB) has been observed and odour-evoked electrical activity in the MOE is reduced in obesity. Obesity also leads to a reduction in OB volume. This is accompanied by a decreased number of neurons surrounding the glomeruli and reduced odour-induced activity in these regions. Both glucose uptake and proinflammatory markers are increased in the OB of obese rodents. Mitral cells, OB neurons that innervate glomeruli and transmit odour information to higher brain areas, are modulated by circulating metabolic hormones; however, in obesity this hormonal regulation becomes dysregulated, further emphasising the bidirectional link between olfactory processing and metabolic state. This figure is available as part of a downloadable slideset

Studies using genetic and DIO models have identified various molecular changes in the MOE that may contribute to olfactory dysfunction in type 2 diabetes and obesity (Fig. 2).

#### Anatomy of OSNs

The cilia of OSNs, which contain olfactory receptors and mediate odorant detection, may be particularly vulnerable to metabolic dysfunction, as mutations in cilia signalling genes have been associated with the development of obesity, and the number and length of neuronal cilia in the brain are reduced with HFD [60]. Consumption of a Western-style diet has been shown to shorten the length of cilia in the MOE [61]. HFD consumption decreases expression of olfactory receptors and the Gα_olf_ protein, a key protein involved in olfactory signal transduction, in the OSNs of mice [48]. Notably, HFD-fed *Kv1.3* knockout mice do not show these changes in olfactory receptor expression, probably because of higher baseline olfactory receptor expression and increased cilia abundance [48, 62]. Because of their exposed location within the MOE, the cilia of OSNs may be susceptible to systemic metabolic disturbances. Structural damage or impaired olfactory receptor expression may significantly disrupt olfactory perception, contributing to the olfactory deficits observed in obesity and type 2 diabetes.

#### MOE homeostasis

The MOE is a highly dynamic structure that is constantly exposed to the external environment and undergoing continuous regeneration [63]. The lifetime of mature OSNs in the MOE has been widely discussed; while the consensus is an average lifetime of 30 days [63, 64], it has been reported that some OSNs can live up to 6 months or even a year [64, 65]. MOE regeneration relies on two stem cell pools [66], with the source depending on whether regeneration is due to homeostatic renewal or tissue repair [66].

High-energy diets alter MOE regeneration, affecting the normal apoptosis and proliferation of OSNs [48, 52, 53]. However, the effect of dietary intervention on MOE homeostasis remains inconsistent across studies. Fructose-enriched diets decrease apoptosis and increase the number of mature OSNs [51], while HFDs often cause OSN loss without modification of MOE thickness and reduction of projections to the OB [48, 67]. However, some studies also report no significant changes in OSN numbers with HFDs [61]. HFD consumption also increases MOE apoptosis [48, 52, 53] and proliferation [48], suggesting accelerated regeneration. Consumption of HFDs also increases the proinflammatory response of the MOE, with a higher presence of microglia and macrophages [48].

In rodents, the MOE is divided into multiple zones, with each characterised by OSNs expressing a specific subset of olfactory receptors [68]. MOE regeneration is not uniform; while the ventral zone is more proliferative [65, 69], the dorsal region shows higher survival rates of OSNs [65]. The dorsal zone is also more sensitive to physiological challenges such as exercise or energy restriction, altering MOE cell dynamics [70] by increasing progenitor proliferation but reducing the number of mature OSNs [70], likely due to an increase in OSN apoptosis [70]. Interestingly, zone-specific alterations triggered by high-energy diets have also been reported [48] with differential outcomes in the different subsets of OSNs [48, 51, 61], probably due to the variable experimental conditions used in these studies. These findings suggest that obesity and insulin resistance selectively impact distinct subsets of OSNs. Genetic models of obesity further highlight the role of diet. *MC4R* knockout mice do not show OSN loss [49], suggesting that OSN degeneration in DIO models results from high fat consumption rather than increased adiposity [48]. Beyond OSN number, dietary interventions can alter olfactory signalling gene expression [13, 33]. Moreover, although findings are inconsistent, human studies suggest that obesity may modify nasal airflow, potentially affecting odorant access at the earliest stage of olfactory processing [71, 72]. Overall, evidence indicates that MOE homeostasis is highly sensitive to dietary influences. These alterations, which vary by zone and OSN subtype, may underlie the olfactory impairments observed in obesity and type 2 diabetes, with implications for metabolic regulation and behaviour.

#### Hormones

The MOE expresses receptors for various hormones linking energy balance to olfaction [10, 73]. Consumption of an HFD has been shown to decrease insulin receptor expression without affecting leptin receptors in obese-prone rats [53]. Both hormones increase spontaneous OSN excitability but decrease odorant-induced activity, potentially reducing the signal-to-noise ratio and impairing olfactory sensitivity [74, 75]. Leptin and insulin levels are lowered in fasting, which may explain improved olfactory thresholds in fasted mice [74, 75]. Insulin also regulates MOE homeostasis by modulating apoptosis and increasing OSN survival [76, 77]. In the streptozocin-induced mouse model of diabetes, insulin deficiency reduces olfactory detection, increases apoptosis and reduces the number of mature OSNs [76], with intranasal insulin administration reversing these effects [76]. Conversely, loss of IGF-1 receptors increases the proliferation of immature OSNs, improving olfaction [59]. Overall, although obesity alters hormonal sensitivity in the MOE, further research is needed to clarify whether disruptions in OSN homeostasis directly drive the development or persistence of obesity.

#### Transporters and fatty acid sensing

The MOE expresses proteins that transport metabolites across various cell types such as OSNs, including glucose transporters (GLUTs) and monocarboxylate transporters (MCTs) [78]. While OSNs primarily express GLUT1 [78], decreased expression of MCT1, GLUT3 and GLUT4 has been observed in the MOE of obese-prone rats [53]. Additionally, a subset of OSNs expresses the fatty acid receptor CD36 linking olfaction to fat detection [79]. However, the impact on olfaction and metabolism of these transporters and receptors, and their alterations in obesity and type 2 diabetes, remains unclear.

### Olfactory bulb alterations in obesity and type 2 diabetes

The OB is the first relay station in the olfactory system, refining sensory signals from the nose before relaying them to higher brain regions. In the context of obesity, this finely tuned structure undergoes significant changes that may affect olfactory perception (Fig. 2).

#### OB anatomy

Obesity affects OB anatomy in both humans and rodents, including reductions in OB volume [29, 80] and OSN projections following exposure to an HFD [48, 67]. However, it is unclear whether this alteration is the result of OSN loss or impaired long-range axonal projection. Furthermore, isopropyl tiglate-induced activation (a ligand for the M72 receptor) is altered in specific regions of the OB in mice fed an HFD, with decreased cell activity in lateral M72 glomeruli and reduced cell counts around medial M72 glomeruli [81]. This specificity also applies to OB interneuron populations, which are essential for odour processing [82]. Consumption of an HFD selectively decreases the number of parvalbumin-expressing interneurons while sparing calbindin-expressing interneurons and doublecortin-expressing immature neurons, which are markers of adult neurogenesis [50], suggesting that neurogenesis itself may remain intact while specific neuronal subtypes are more susceptible to changes related to an HFD. Furthermore, elevated levels of proinflammatory markers have been identified in the OB [83, 84], potentially contributing to structural and functional alterations in olfactory processing.

#### Hormones

Here, we consider specific hormones involved in the metabolic regulation of the OB. See reviews by Stark, Faour et al, Guzmán-Ruiz et al and Palouzier-Paulignan et al for more on the neuroendocrine functions of the OB [8–11].

#### *Ghrelin*

During food deprivation, elevated levels of hormones such as the orexigenic stomach-derived hormone ghrelin increase OB activity, thereby increasing sensitivity to food-related odours [8–11]. Interestingly, deletion of ghrelin receptors in the OB not only leads to reduced olfactory sensitivity in mice but also altered feeding behaviour, body composition and glucose homeostasis [85], linking ghrelin-sensitive neurons in the OB to both olfaction and energy balance. In DIO, reduced ghrelin levels and impaired transport to the OB correlate with reduced olfactory sensitivity and odour-evoked brain activity [86–90].

#### *Insulin*

Insulin, which is released in response to food perception and postprandial increases in blood glucose levels, is a key regulator of OB activity. This is emphasised by its rapid transport across the blood–brain barrier [91] and the high density and affinity of insulin receptors in the OB [10, 11]. In rodents, insulin has been shown to reduce olfactory performance [90, 92, 93]. Electrophysiological studies have revealed that acute insulin exposure increases mitral cell (MC) firing rates, whereas chronic exposure suppresses activity [57]. In DIO, insulin resistance impairs this modulation of MCs by insulin, which is further exacerbated by reduced insulin transport across the blood–brain barrier [10]. The role of the OB in energy homeostasis is further highlighted by studies on the insulin-sensitive potassium channel Kv1.3, which have shown that *Kv1.3* function in the OB is required to protect lean mice from DIO [49, 94, 95], and blocking Kv1.3 function reduces bodyweight by altering feeding behaviour in obese mice [95].

#### *Glucagon-like peptide-1*

Glucagon-like peptide-1 (GLP-1) is released postprandially from the small intestine in response to nutrient ingestion, with its release altered inconsistently in obesity and diabetes [96, 97]. GLP-1 enhances olfactory sensitivity by increasing MC excitability [98]. Interestingly, preproglucagon, a GLP-1 precursor, is expressed in OB interneurons, which suggests that GLP-1 is released locally in the OB [99]. GLP-1 analogues, such as exendin-4, have been shown to improve olfactory performance in DIO rodents [98, 100] and enhance cephalic phase insulin release during food perception [101–103], dependent on GLP-1 receptors in the OB [100], further linking the role of GLP-1 in feeding behaviour to its actions in the olfactory system. The potential of GLP-1 analogues to target the OB when administered intranasally or intraperitoneally [98, 101] suggests that the olfactory pathway is a promising therapeutic avenue in obesity and type 2 diabetes.

#### *Leptin*

Leptin, which is released from adipocytes in proportion to body fat mass [104], inhibits MC activity, reducing food odour exploration, as seen in leptin-deficient mice, which exhibit increased olfaction that is normalised by leptin administration [9, 11]. While DIO increases levels of circulating leptin [84, 104], it also induces leptin resistance, a state with diminished sensitivity to rising leptin levels [105, 106]. Whether the OB is similarly subject to this resistance, and whether it could affect olfactory performance, remains to be fully elucidated.

#### Glucose sensing

Olfactory processing is a high-energy process [107] that relies heavily on glucose transport across the particularly permeable blood–brain barrier in the OB [10]. MCs in the OB are capable of sensing glucose and exhibit key characteristics of glucose-sensing cells, including the expression of several GLUTs [11], and MC activity can be modulated by rising glucose levels [108]. Interestingly, while brain glucose uptake is impaired in both DIO and type 2 diabetes [67, 109–111], DIO mice exposed to an HFD show increased glucose metabolism in the OB [112], further illustrating the complex relationship between glucose metabolism, olfaction and obesity.

#### Fatty acid sensing

In addition to glucose, mitral and tufted cells (MTCs) in the OB also sense fatty acids [113, 114], synchronising their activity during odorant detection and potentially enhancing olfactory precision [115]. Notably, the OB has high levels of GPR40, a G-protein-coupled receptor for NEFAs. On ligand binding, GPR40 activation triggers calcium influx, which may modulate MTC excitability and, consequently, influence odour processing [116].

### Impact of metabolic dysfunction on olfactory pathways and downstream targets

Obesity and type 2 diabetes are associated with widespread changes not only in the MOE and OB, but also in the piriform cortex. Notably, human studies have shown that activity in the piriform cortex is associated with the ability to imagine odours, which in turn correlates with feeding behaviour and indirectly predicts changes in BMI and body fat percentage [32, 117], thereby linking piriform cortex function to the development of obesity. Rodent studies have further demonstrated that the piriform cortex integrates metabolic signals to regulate olfactory function and energy balance. Superficial pyramidal neurons in this region express the insulin-sensitive potassium channel Kv1.3, whose activity is modulated by insulin under conditions of high glucose, impairing olfactory discrimination in lean rats [118]. The loss of *Kv1.3* enhances the activity of superficial pyramidal neurons in the piriform cortex, improves olfactory performance and alters feeding behaviour [119]. Conditional knockout of *Kv1.3* in the piriform cortex reduces bodyweight and increases food intake following fasting [119], further underscoring the role of Kv1.3 signalling in the olfactory pathway as a critical mediator of whole-body metabolism. Interestingly, a study in DIO mice reported no significant differences in neuronal activation, neurogenesis or piriform cortex size compared with lean control mice [46]. While some studies have found no major structural changes in the piriform cortex in obesity [120], treatment with a GLP-1 receptor agonist has been shown to reverse cellular stress in a rat model of diabetes [120], suggesting therapeutic potential.

## The emerging role of olfaction in the regulation of metabolism in rodents

The olfactory system is not merely a sensory modality but actively regulates whole-body metabolism, highlighting a bidirectional relationship between olfaction and metabolic control. Here, we discuss the olfactory regulation of whole-body metabolism primarily based on rodent studies; for a detailed examination of human data see Li et al [121].

### Sensory regulation of energy and glucose homeostasis

Food sensory cues modulate hypothalamic neurons critical for maintaining energy and glucose homeostasis [122–126]. Several hypothalamic neuronal populations, including the orexigenic agouti-related peptide (AgRP)-expressing and anorexigenic pro-opiomelanocortin (POMC)-expressing neurons in the arcuate nucleus (ARC), modulate their activity in response to food-related sensory cues, that is, sight, smell and learned predictive signals [122–126], with olfactory cues being sufficient to modulate both AgRP and POMC neuronal activity [123, 127]. This sensory-driven modulation of hypothalamic neuronal activity aligns with postprandial responses, suggesting a coordinated and anticipatory regulatory mechanism by which the hypothalamus dynamically integrates sensory and metabolic signals to adaptively modulate feeding behaviour. Our recent study demonstrated that food odours elicit a widespread brain activation extending beyond the olfactory cortex and hypothalamus [128]. Notably, food odours activate glutamatergic neurons in the medial septum, and stimulating the OB projections to this region suppresses feeding in lean but not DIO mice [128], suggesting that obesity disrupts the olfactory modulation of feeding behaviour and whole-body metabolism. Further supporting the link between obesity and disrupted sensory modulation, the inhibition of AgRP neuronal activity in response to sensory food cues is markedly blunted in DIO mice and partially restored after weight loss [129]. Whether this disruption is due to altered olfactory processes, impaired signalling within olfactory–metabolic circuits or intrinsic changes in downstream neuronal populations remains to be clarified.

Importantly, food sensory cues trigger several peripheral metabolic responses prior to food consumption, for example hepatic mTOR activation, brown adipose tissue thermogenesis and changes in lipid metabolism [130–133], preparing the body for nutrient intake. However, the selective role of sight or smell in this regulation needs to be further elucidated. DIO affects these olfactory-induced metabolic responses, impairing lipid metabolism in response to food odours [131]. However, repeated food odour exposure under intermittent fasting improves glucose metabolism in DIO mice, suggesting that sensory modulation has potential therapeutic applications in managing obesity [131]. The sensory perception of food also triggers rapid insulin secretion, known as cephalic phase insulin release. Multisensory food cues, such as combined olfactory, gustatory and visual signals, robustly induce cephalic phase insulin release in lean individuals [134] but this is impaired in human [135] and rodent [100, 103, 135] studies of obesity. Montaner et al have further demonstrated that GLP-1 receptor activation in the OB of lean mice increases cephalic phase insulin release, but this mechanism is impaired in DIO mice [103], underscoring the importance of sensory input in feeding behaviour and its potential role in obesity management. Targeting these disrupted pathways may offer novel strategies for therapeutic intervention in metabolic diseases.

### Sensory regulation of feeding-related behaviour

In rodents, olfactory perception of food-related odours can either increase [61, 119, 136] or have no effect on [137] subsequent feeding behaviour, with findings varying based on odour type, metabolic state and experimental design. Interestingly, a recent study demonstrated that prolonged exposure to food odours decreases feeding [138]. Exposure to non-food-related odours, such as an aversive predator odour, influences DIO and whole-body metabolism with sex-specific phenotypes [139, 140]. Taken together, these findings underscore the potent and context-dependent role of olfaction in shaping feeding behaviour and energy balance, which may be disrupted in obesity.

The sensory regulation of feeding behaviour has also been explored in humans [3, 141], with similarly mixed and sometimes contradictory outcomes. While several studies report increased food consumption following odour exposure [142–147], others have found no significant changes in food intake [141, 148–152]; and a few studies have even reported a reduction in food consumption [141, 153, 154]. Similarly, evidence regarding the impact of odours on food intake in individuals with obesity is limited and inconsistent. While some studies report no significant effect on food intake [148, 152], others observe an increase in consumption following odour exposure [144, 155]. Beyond effects on food consumption, odour exposure has been shown to affect food preferences and appetite in both individuals without [147, 156–159] and with [155, 160, 161] obesity. Crucially, human studies have shown that bypassing the cephalic phase of food consumption by delivering nutrients directly into the stomach does not fully suppress appetite [162–164], while reintroducing sensory stimulation, by allowing individuals to chew food without swallowing during enteral feeding, significantly enhances feelings of fullness [162]. This strongly suggests that post-ingestive nutrient sensing alone is insufficient for generating satiety and that sensory cues, particularly those related to taste and smell, play an essential role in regulating appetite and satiety in humans.

## Olfactory system-based therapeutics

**Fig. 3:**
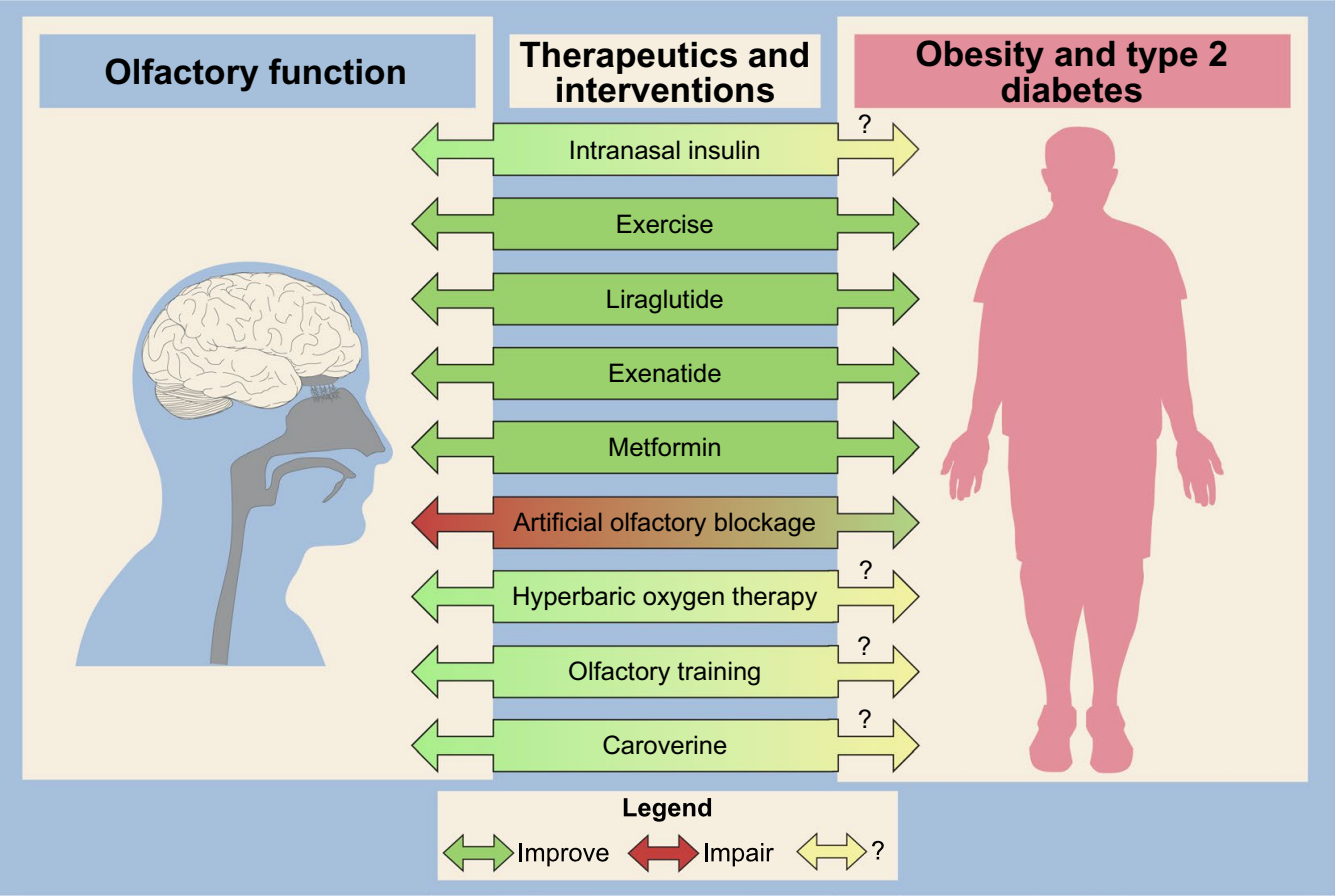
The olfactory system as a therapeutic target in metabolic disease. The potential bidirectional relationship between olfactory function and obesity and type 2 diabetes suggests that restoring or enhancing olfactory function may represent a therapeutic strategy for obesity and type 2 diabetes. Supporting this idea, some treatments already used in clinical settings have been shown to modulate olfactory performance. For example, the glucose-lowering and anti-obesity drugs liraglutide, exenatide and metformin have been associated with improved olfactory function (green). Similarly, intranasal insulin, caroverine, olfactory training and hyperbaric oxygen therapy enhance olfactory sensitivity, although their exact metabolic effects remain unclear (yellow). In addition, while intranasal blockade of the olfactory system leads to an impaired sense of smell (red) but improves metabolism (green), exercise improves both metabolism and olfactory function. Collectively, these findings open a novel therapeutic avenue, whereby targeting the olfactory system could influence metabolic outcomes and provide dual benefits in the treatment of metabolic disease. This figure is available as part of a downloadable slideset

Beyond impairing quality of life, olfactory dysfunction observed in obesity and type 2 diabetes may contribute to the progression of disease. Restoring olfactory function could therefore provide dual benefits, improving both metabolic outcomes and sensory performance. Moreover, olfactory decline also occurs with ageing and serves as an early marker of neurodegenerative and psychiatric disorders [165, 166], broadening therapeutic implications. Over the past decade, various treatment strategies have been explored to improve olfactory dysfunction [167]. Among these treatment strategies, intranasal drug delivery has emerged as a promising, non-invasive method to target olfactory structures [9] involved in hormonal and metabolic signalling [10] (Fig. 3). However, intranasally administered compounds may also reach other brain regions or peripheral tissues, which may result in additional physiological effects [168].

### Intranasal insulin

Although systemic insulin resistance is linked to reduced olfactory function in both humans [18] and rodent models [56], and central intracerebroventricular insulin administration acutely decreases olfactory detection in fasted rats [169], recent evidence points to intranasal insulin as a potential therapeutic approach for improving olfactory function in humans [167, 170, 171]. Intranasal insulin administration enhances olfactory performance in a dose-dependent manner [172]. Findings in healthy individuals are variable [172–174], with one study reporting increased activation in olfactory–visual integration areas [174], and another observing reduced sensitivity to a non-food odour [173]. However, further research is needed to determine the optimal insulin dose and duration and method of insulin administration [167, 170, 171]. While rodent studies strongly support an active role for insulin within the olfactory system [169, 175], additional investigation is required to better define its specific contribution to the olfactory system compared with other brain areas in humans.

### Other pharmacological treatments

Beyond insulin, other drugs have been studied for their potential effects on olfactory function. Metformin reduces olfactory dysfunction in individuals with diabetes [176], and GLP-1 receptor agonists, such as liraglutide and exenatide, improve odour identification in individuals with obesity with type 2 diabetes [167]. Caroverine, a non-competitive ion channel antagonist, has also been reported to enhance olfactory function [167]. Rodent studies have provided additional insight into pharmacological interventions. In Goto–Kakizaki rats, linagliptin, a dipeptidyl peptidase-4 inhibitor, failed to improve olfactory deficits [56].

### Alternative treatments

Beyond pharmacological interventions, alternative treatments have been studied for their potential to improve olfactory function in humans, including olfactory training improving odour discrimination [167] and hyperbaric oxygen therapy [167]. Emerging evidence also suggests that physical activity may improve olfactory function by improving odour thresholds, identification and intensity [177]. Similar findings have been observed in adolescent mice fed an HFD, where exposure to an enriched environment combining social stimulation, physical exercise and sensory training restored normal odour sensitivity and olfactory memory [50]. Conversely, in humans, preliminary data have shown that reducing olfactory sensitivity with silicone inserts improves insulin sensitivity, reduces sweet preference and promotes weight loss in younger individuals [178]. Collectively, these findings indicate that several approaches aimed at enhancing olfaction might also improve metabolism in obesity and type 2 diabetes; however, further research is needed to clarify the mechanisms involved and the beneficial long-term effects of these interventions. Furthermore, it remains uncertain whether these metabolic benefits are directly mediated by improved olfactory function or indirectly through changes in behaviour. Restoration of smell may influence food choice and dietary habits [3], or other lifestyle factors [179] that could contribute to improved metabolic outcomes, highlighting the need for future studies to clarify the underlying mechanisms.

## Olfactory dysfunction in obesity and type 2 diabetes: cause, consequence or both?

Recent studies challenge the view that olfactory dysfunction in obesity and type 2 diabetes is merely a secondary effect, instead pointing to a bidirectional relationship whereby olfactory dysfunction appears to be both a consequence of and a contributor to metabolic disease. Clinical and preclinical work consistently link obesity and diabetes with altered olfaction, and rodent studies show that targeted manipulations of the olfactory system can influence whole-body metabolism and obesogenic responses. These findings position the olfactory system as an active regulator of energy and glucose homeostasis, yet the temporal dynamics and underlying mechanisms remain unclear. Moving forward, uncovering the precise mechanisms underlying olfactory dysfunction in obesity and type 2 diabetes may allow the development of innovative olfaction-based interventions for obesity and metabolic disorders.

## Supplementary Information

Below is the link to the electronic supplementary material.

Slideset of figures (PPTX 516 KB)

## Abbreviations

AgRP: Agouti-related peptide
DIO: Diet-induced obesity
GLP-1: Glucagon-like peptide-1
GWAS: Genome-wide association study
HFD: High-fat diet
MC: Mitral cell
MOE: Main olfactory epithelium
MTCs: Mitral and tufted cells
OB: Olfactory bulb
OSN: Olfactory sensory neuron
POMC: Pro-opiomelanocortin
