# Supplementary material for: Olfactory dysfunction in obesity and type 2 diabetes: mechanistic insights from preclinical models
Source: Diabetologia. 2026 May 30;69(8):2114–30. doi: 10.1007/s00125-026-06755-w (PMC13310233; doi:10.1007/s00125-026-06755-w)
Supplement: Supplementary file 1 — Slideset of figures (PPTX 516 KB) [file 125_2026_6755_MOESM1_ESM.pptx]

## Slide 1
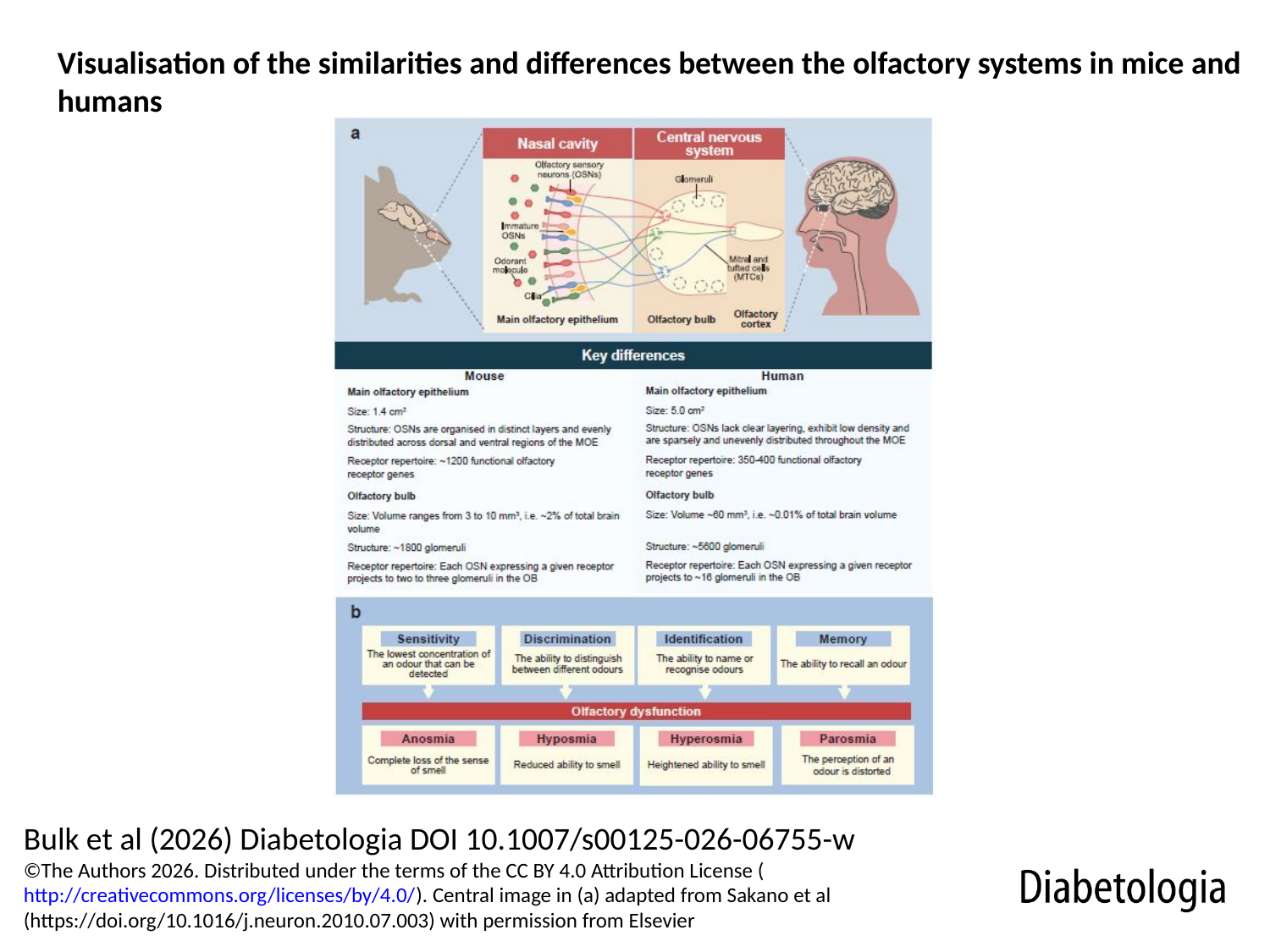

Visualisation of the similarities and differences between the olfactory systems in mice and humans
Bulk et al (2026) Diabetologia DOI 10.1007/s00125-026-06755-w
©The Authors 2026. Distributed under the terms of the CC BY 4.0 Attribution License (http://creativecommons.org/licenses/by/4.0/). Central image in (a) adapted from Sakano et al (https://doi.org/10.1016/j.neuron.2010.07.003) with permission from Elsevier

## Slide 2
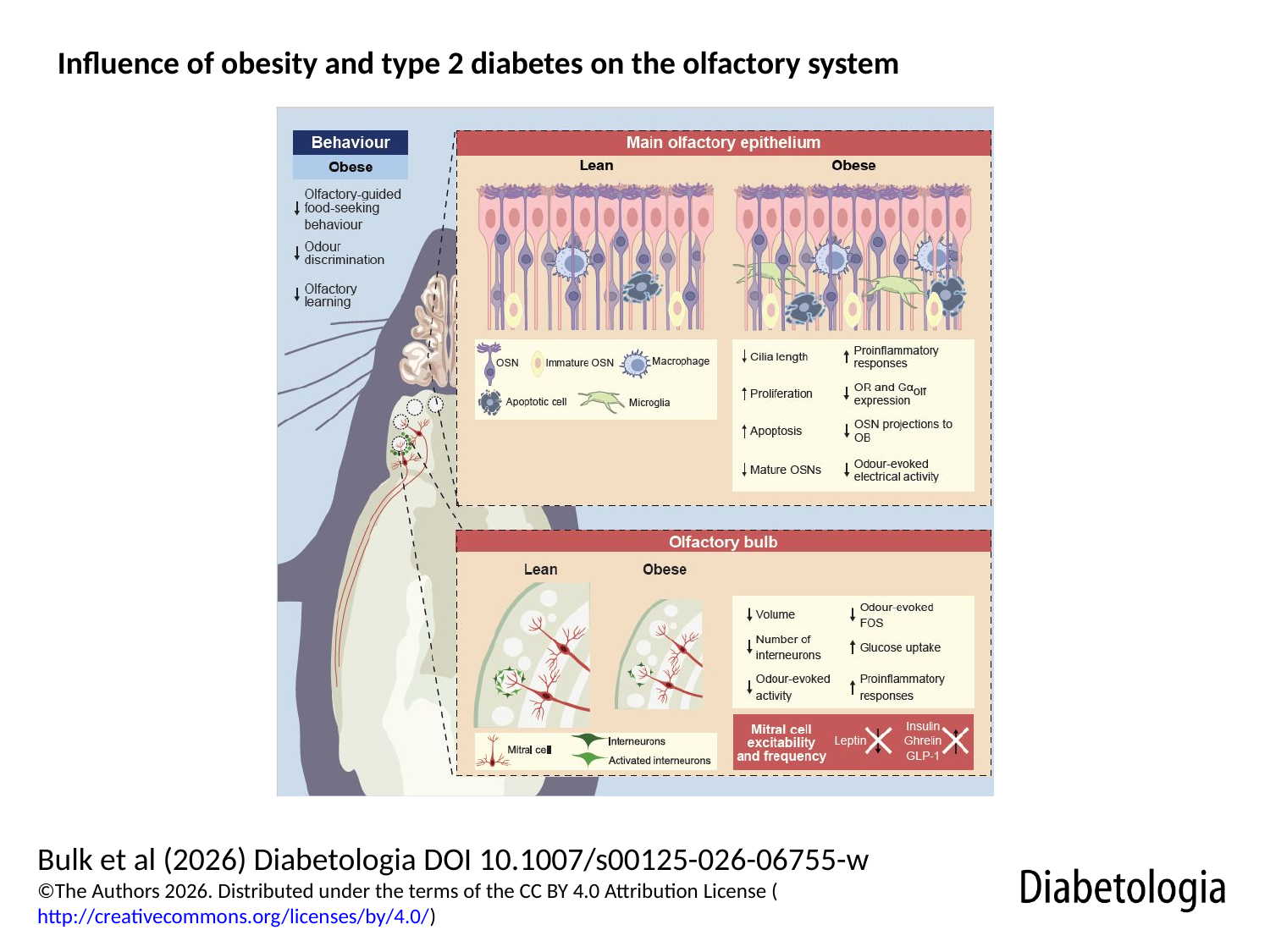

Influence of obesity and type 2 diabetes on the olfactory system
Bulk et al (2026) Diabetologia DOI 10.1007/s00125-026-06755-w
©The Authors 2026. Distributed under the terms of the CC BY 4.0 Attribution License (http://creativecommons.org/licenses/by/4.0/)

## Slide 3
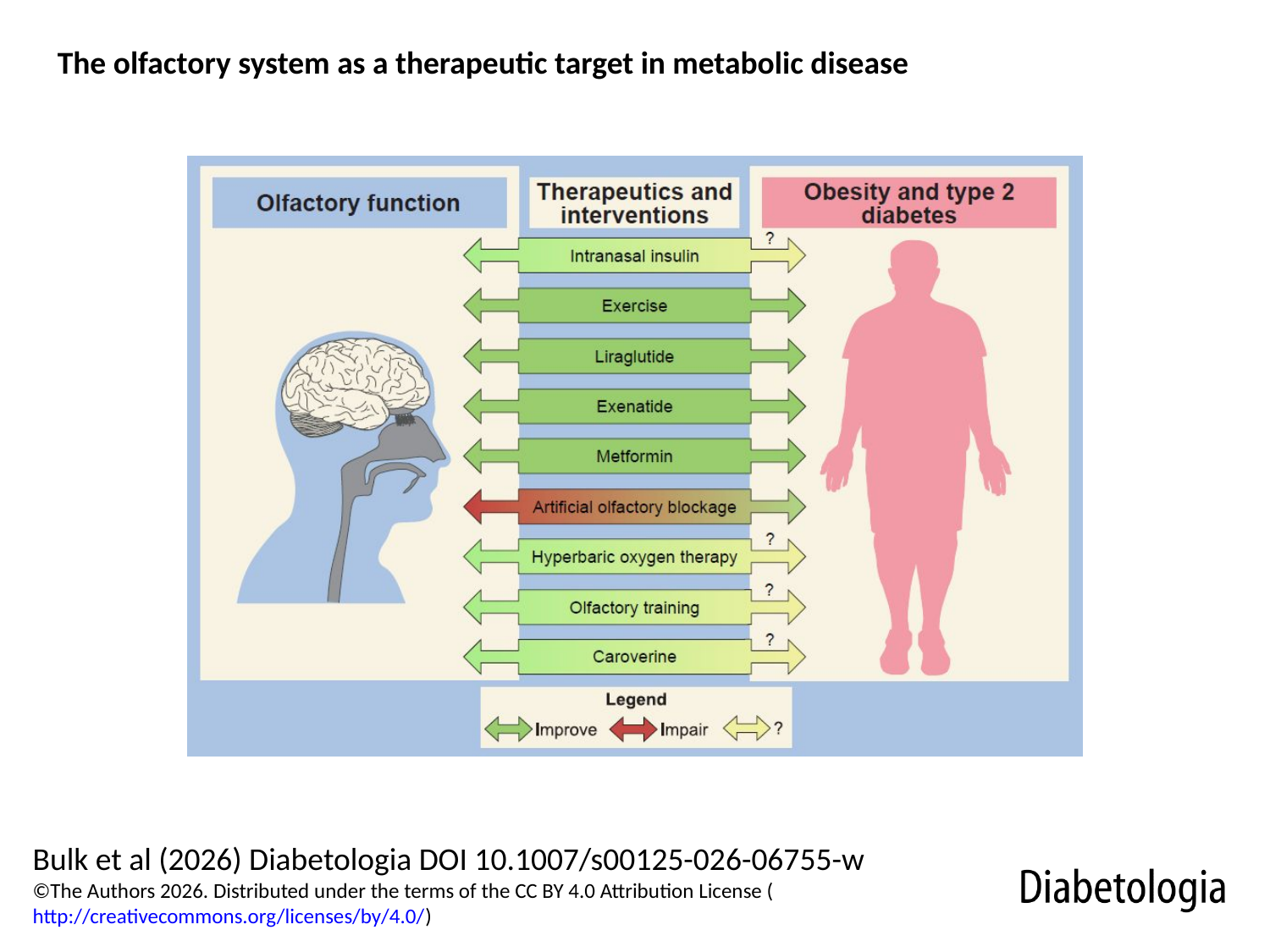

The olfactory system as a therapeutic target in metabolic disease
Bulk et al (2026) Diabetologia DOI 10.1007/s00125-026-06755-w
©The Authors 2026. Distributed under the terms of the CC BY 4.0 Attribution License (http://creativecommons.org/licenses/by/4.0/)
